# The learning curve of laparoscopic single-site total hysterectomy with conventional laparoscopic instruments

**DOI:** 10.1097/MD.0000000000045805

**Published:** 2025-11-28

**Authors:** Jun Wu, Jin Zhao, Jin Dong, Zhengwei Lai

**Affiliations:** a The First People’s Hospital of Xiaoshan District, Xiaoshan Affiliated Hospital of Wenzhou Medical University, Hangzhou, Zhejiang, China.

**Keywords:** conventional laparoscopy (CL), cumulative sum (CUSUM) analysis, single-port laparoscopy (SL)

## Abstract

Total hysterectomy is a relatively intricate surgical procedure in clinical practice. Laparoscopic total hysterectomy has become widely adopted in clinical settings. With the clinical advancement of single-port technology, single-port laparoscopy (SL) has garnered extensive popularity due to its safety and aesthetic appeal. This method involves entering the abdominal cavity through a single incision at the umbilicus, offering benefits such as minimal trauma, rapid recovery, and improved cosmesis. However, the high operational complexity results in a longer learning curve. This study retrospectively analyzed cases of laparoscopic total hysterectomy performed at the First People’s Hospital of Xiaoshan District, Hangzhou City, from January 2020 to October 2024. The analysis included 50 cases, with 25 conducted via SL and 25 via conventional 3-port laparoscopy (CL). Various parameters were collected, including body mass index, uterine size, operative duration, changes in hemoglobin levels, leukocyte levels, neutrophil levels, C-reactive protein levels, and patient fever incidence. The cumulative sum (CUSUM) technique was employed for learning curve analysis to assess surgical proficiency. CUSUM analysis is a statistical method to monitor cumulative deviations from a target value, was used to objectively quantify the learning process by tracking operative time trends across sequential cases. This study showed that there were no statistically significant differences in the baseline characteristics and postoperative changes between the 2 groups. CUSUM analysis revealed a proficiency threshold at 12 cases, after which SL operative time (123 ± 15 min) matched CL (118 ± 14 min; *P* = .32). In total hysterectomy procedures, once proficiency is achieved, SL can yield safety outcomes comparable to those of CL. These findings provide valuable guidance for gynecologists interested in adopting SL. As technology continues to evolve and experience accumulates, SL is poised to offer patients an increasing array of minimally invasive treatment options.

## 1. Introduction

Total hysterectomy is a prevalent clinical procedure for treating common gynecological benign tumors such as uterine fibroids and adenomyosis.^[1]^ Hysterectomy is among the most frequently conducted gynecological procedures. The first instance of a laparoscopic hysterectomy was documented in 1989.^[2]^ Single-port laparoscopy (SL), recognized for its minimal postoperative scarring and enhanced cosmetic outcomes, is gaining popularity among women opting for total hysterectomy.^[3,4]^ The most prominent benefit of LESS lies in the extremely small number of incisions. This characteristic significantly reduces the invasiveness of the surgical procedure compared to traditional methods.^[5]^ The minimal number of incisions not only results in less tissue trauma but also contributes to a faster postoperative recovery for patients.^[6]^ Moreover, it often leads to less scarring, which can have positive psychological impacts on patients. Recent meta-analyses confirm SL’s safety.^[7]^ Despite this, the intricacy of SL and the absence of surgical triangulation, along with the “chopstick effect” where operating instruments can interfere with each other and the restricted surgical view, increase the procedure’s difficulty. Even seasoned laparoscopic surgeons need considerable time to refine their techniques, limiting the widespread clinical application of this technology and underscoring the clinical importance of defining its learning curve. To date, there is a dearth of literature on the learning curve associated with SL in total hysterectomy. This retrospective study examines 50 cases of laparoscopic hysterectomies performed by the same principal surgeon at the First People’s Hospital of Xiaoshan District, Hangzhou, from June 2020 to June 2022. Clinical data were meticulously documented, and the learning process was quantified using cumulative sum (CUSUM) analysis to deduce the learning curve. The aim is to offer a reference for gynecologists learning single-port laparoscopic surgery.

## 2. Materials and methods

This study conducted a retrospective analysis of laparoscopic total hysterectomy cases performed at the First People’s Hospital of Xiaoshan District, Hangzhou, from June 2020 to June 2022. The study included 25 cases performed using SL and 25 cases using conventional laparoscopy (CL). A single surgeon conducted all operations, and all cases involved total hysterectomy. The parameters collected encompassed body mass index (BMI), uterine size (described by analogy to the size of the uterus in pregnancy months), surgery time, changes in hemoglobin levels, postoperative fever incidence, leukocyte count variations, neutrophil count shifts, and postoperative C-reactive protein levels. The inclusion criteria were as follows: patients in good general health, no contraindications to surgery identified preoperatively, stable vital signs, BMI ≤ 35 kg/m^2^, no previous history of abdominal surgery or malignant tumors. Written informed consent for the surgery was obtained from patients by the First People’s Hospital of Xiaoshan District. The Ethics Committee of the First People’s Hospital of Xiaoshan District has approved the study.

## 3. Surgical techniques

**Figure 1.**
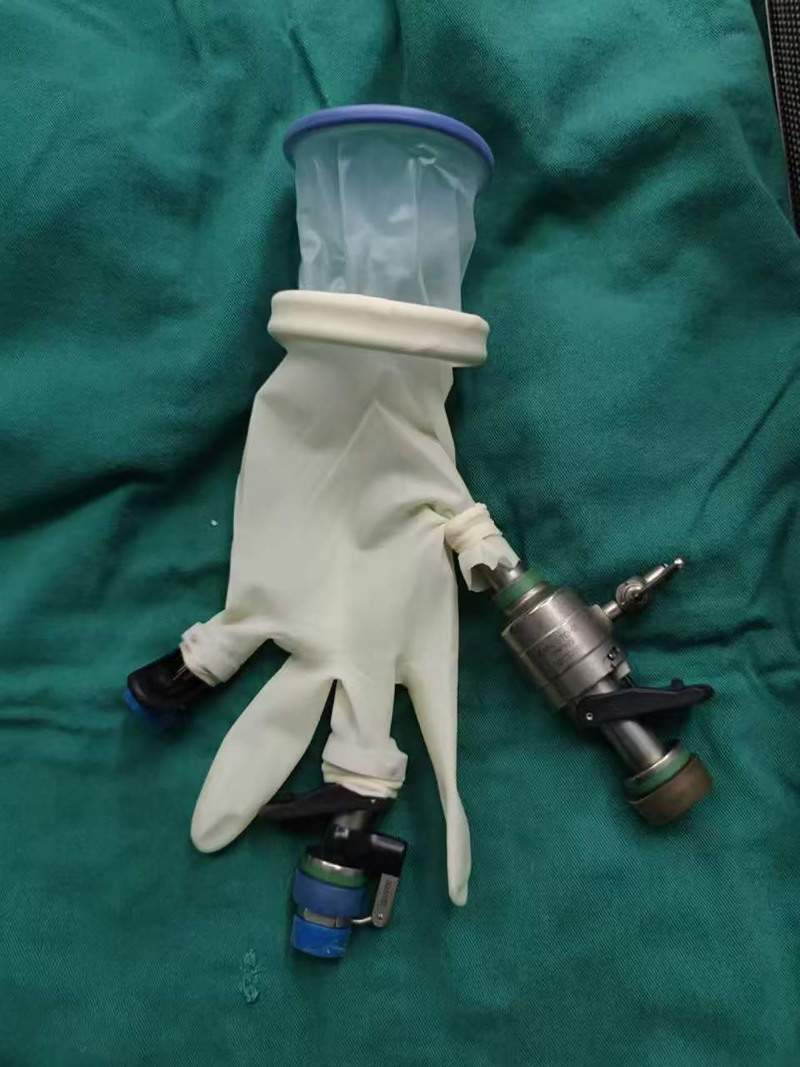
The homemade glove port.

**Figure 2.**
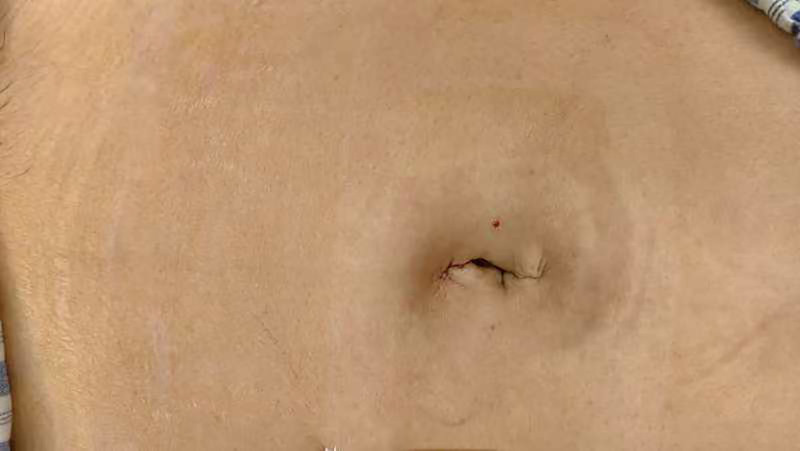
Display after suturing the umbilical wound after surgery.

The operations were all conducted under general anesthesia with the patient positioned in lithotomy and a uterine manipulator in use. For SL, the same instruments as in CL were utilized, comprising an ultrasonic scalpel, bipolar coagulation devices, size 0 Ethibond sutures (Johnson, USA), and a laparoscope (STORZ, Germany). We fabricated a homemade single-port access platform consisting of a size 7 rubber glove coupled with 2 10 mm and one 5 mm trocar (STORZ, Germany; as illustrated in Fig. 1). Guided by the natural skin folds within the umbilicus, a longitudinal incision of 1.5 to 2.0 cm was created at the navel. Through this incision, the homemade glove port was introduced into the abdominal cavity, and 14 kPa of CO_2_ was insufflated to establish a pneumoperitoneum, which facilitated the execution of the total hysterectomy. Postoperative appearance of the umbilical incision is presented in Figure 2. The chopstick effect is minimized by interleaving instruments and adjusting the laparoscopic angle.

## 4. Data analysis

Data analysis was performed using GraphPad Prism. Continuous variables were tested for normality and homogeneity of variance. Normally distributed variables with homogeneous variances were expressed as mean ± standard deviation (*x* ± *s*) and analyzed using *t*-tests. Nonparametric Wilcoxon rank-sum tests were employed to evaluate data that were not normally distributed or had heterogeneous variances. A *P*-value < .05 was considered statistically significant. The learning curve was assessed using the CUSUM method. The formula for CUSUM is given by: $CUSUM=\underset{1=1}{\overset{n}{\sum }}\;T_{i}-T_{m}$, where n represents the number of surgical cases, *T*_*i*_ denotes the time for each individual surgical case, and *T*_*m*_ is the mean surgical time.

## 5. Results

**Figure 3.**
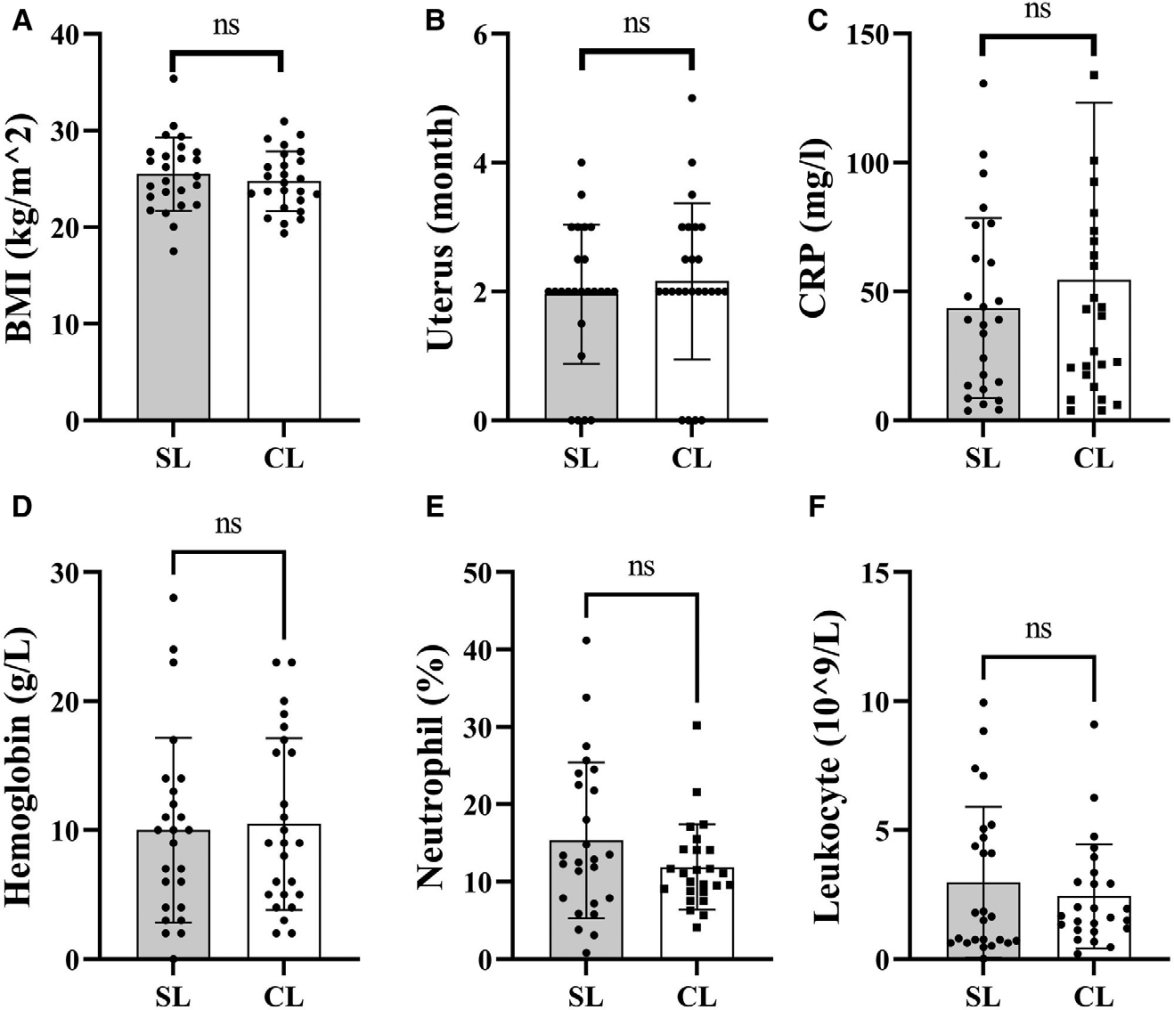
Preoperative and postoperative indices. (A) BMI index; (B) uterus size; (C) CRP; (D) hemoglobin; (E) neutrophil; (F) leukocyte. Each column represents the mean ± SEM, n = 25. BMI = body mass index, CRP = C-reactive protein, ns: no significance.

**Figure 4.**
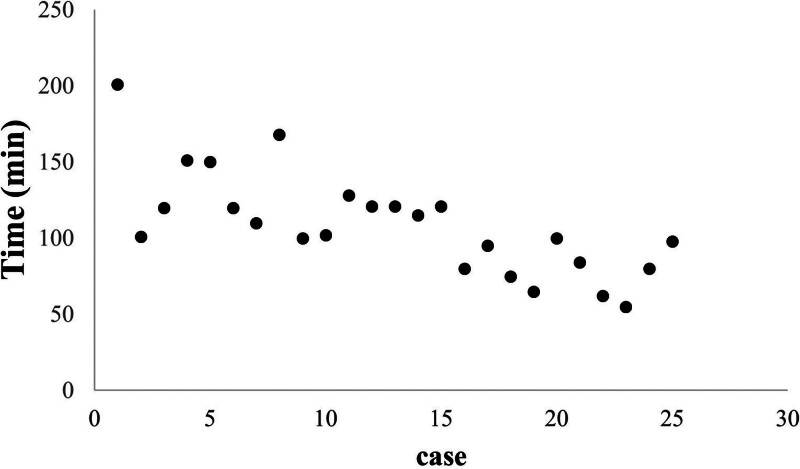
Scatter plot of surgical time for each salpingectomy case.

**Figure 5.**
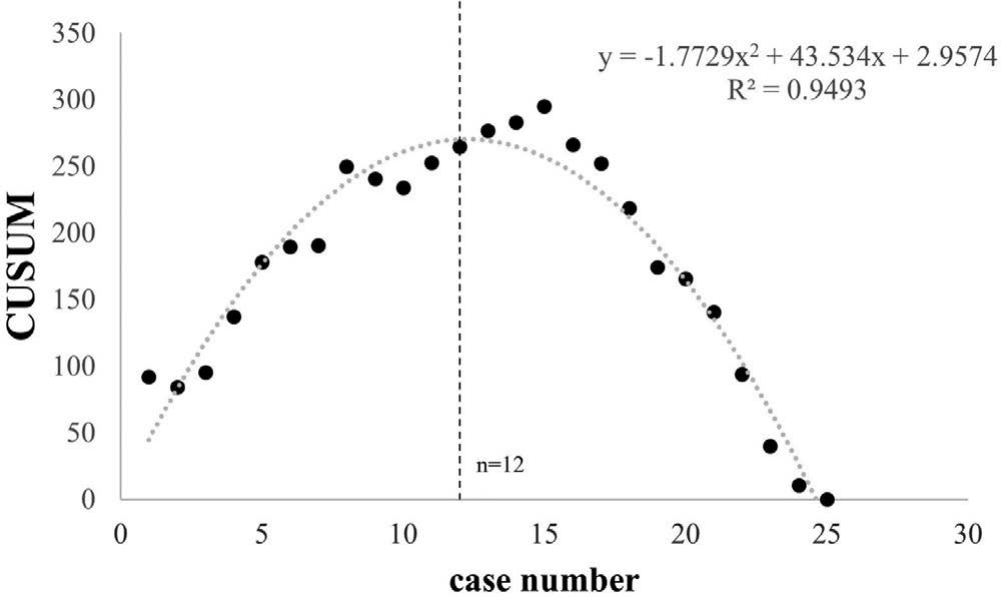
Learning curve of operational time for salpingectomy.

**Figure 6.**
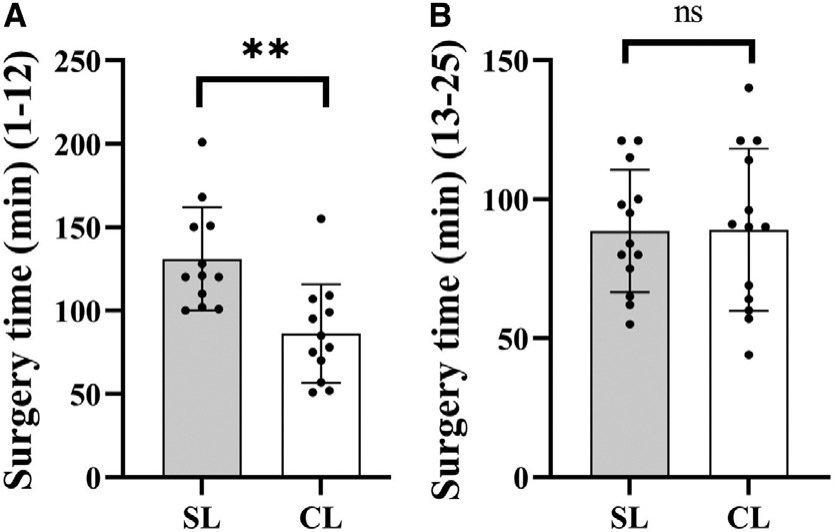
Surgery time. (A) The first 12 cases; (B) after 13 cases. **P < .01. Each column represents the mean ± SEM, n ≥ 12. ns: no significance.

The general characteristics of the 50 patients involved in this study. Twenty-five female patients underwent total hysterectomy via SL, and another 25 via CL. No intraoperative complications occurred, and during the 1-month follow-up period, no cases of umbilical hernia, incisional infection, urinary tract, or gastrointestinal injuries were detected. There were no statistically significant differences between the 2 groups in terms of BMI (*P* = .457) and uterine size (*P* = .541; Fig. 3A, B). The changes in hemoglobin pre- and postoperatively (*P* = .807) were used to reflect intraoperative blood loss, while changes in leukocyte counts (*P* = .455), neutrophil percentages pre- and postoperatively (*P* = .139), postoperative C-reactive protein levels (*P* = .480), and fever incidence (18/25 SL, 20/25 CL; *P* = .576) were used to assess postoperative infection. No statistically significant differences were found in these comparisons (Fig. 3C–F). A scatter plot of surgery time versus case number is depicted in Figure 4, and the CUSUM curve is shown in Figure 5. The curve fit is good with an *R*^2^ value of 0.930. The curve equation in the formula is *Y* = 0.0174*X*^3^ − 1.4188*X*^2^ + 25.5112*X* − 26.8183. The CUSUM curve is generated by calculating the difference between each case’s operative time and the mean operative time, then sequentially accumulating these values to plot corresponding data points. A negative slope in the CUSUM curve indicates that operative times are shorter than the average, reflecting improved surgical proficiency. As the number of cases increased, the surgery time gradually decreased, stabilizing after the 12th case. We found that in the first 12 cases, the surgery time for the SL group was significantly longer than the CL group (*P* = .002; Fig. 6A). However, for the last 13 cases, there was no statistically significant difference in surgery time between the 2 groups (*P* = .964; Fig. 6B).

## 6. Discussion

Total hysterectomy is a common surgical procedure, primarily used for treating patients with large uterine fibroids, severe adenomyosis, and other conditions. Laparoscopic hysterectomy is a widely adopted technique, which, compared to vaginal hysterectomy, has fewer restrictions regarding the size of the uterus and vaginal conditions, making it suitable for the majority of cases. It also carries a lower risk of infection, but this comes with increased costs and trauma.^[8,9]^ Laparoscopy is further divided into CL and SL. Compared to CL, SL is less invasive and more aesthetically pleasing for patients. By using a homemade port, it does not add to the patient’s financial burden. The field of SL procedures is witnessing swift growth, attracting the interest of a growing cadre of surgical professionals who are dedicating their attention to mastering this advanced method.^[10]^ However, SL requires a higher level of proficiency from the surgeon to be effectively performed.

In this study, we compared various data between single-incision and multi-port hysterectomies performed by the same surgeon using conventional laparoscopic instruments within the same time frame. We found that there were no statistically significant differences between the 2 groups in terms of blood loss, leukocyte changes, and neutrophil changes, among other factors, except for the operative time. There were no surgical failures or conversions to multi-port surgery in the single-incision group, and no postoperative complications such as umbilical wound infection or hernia occurred in this group. But our study found frequent transient postoperative fever in patients, necessitating optimized management. SL’s minimal invasiveness accelerates recovery,^[11]^ yet its refinement requires integrating nutritional support,^[12,13]^ complication mitigation,^[14]^ and technological innovation to enhance outcomes.^[15]^.

The mastery of SL is contingent upon navigating unique challenges such as the absence of surgical triangulation, interference among surgical instruments, and limited visual fields, which together demand a distinct learning curve. The CUSUM analysis technique provides a quantitative measure of this learning process, facilitating the gradual acquisition and refinement of specific surgical skills through ongoing education.^[16]^ This method is typically assessed based on the number of surgical cases required to achieve a level of proficiency in surgery.^[17,18]^ Within the realm of gynecology, single-incision laparoscopic procedures exhibit varying learning curves for different techniques and procedures. For instance, proficiency in single-port laparoscopic salpingo-oophorectomy is generally achieved after 10 to 15 cases,^[5]^ while single-port laparoscopic myomectomy requires up to 45 cases to surmount the learning curve.^[19]^ Single-incision laparoscopic salpingectomy can be mastered in approximately 11 cases.^[20]^ Our study indicates that proficiency in performing total hysterectomy via SL is attainable after approximately 12 cases, with a subsequent significant reduction in operative time. Comparative analysis revealed a notable difference in operative time during the initial 12 cases; however, in the subsequent 13 cases, there was no statistically significant difference in operative time between the SL and CL groups. The threshold in 12 cases may be related to the prior CL experience of the surgeon, which shortened the SL adaptation period.

A limitation of this study is that the principal surgeon concurrently performed other procedures via SL, such as salpingectomy and oophorectomy, during the same period. This concurrent practice effectively accelerated the surgeon’s proficiency, which may have influenced the study’s outcomes. Future studies with a single procedure are needed.

## 7. Conclusion

Utilizing conventional laparoscopic instruments, CUSUM analysis indicates that proficiency in performing total hysterectomy via SL can be achieved after approximately 12 cases, with operative times subsequently reduced to a level indistinguishable from those of CL. Moreover, no significant differences were observed between the 2 approaches regarding intraoperative blood loss and postoperative complications. SL achieved the same safety and efficiency as CL after 12 cases. It is suggested that SL should be included in the gynecological minimally invasive training system. Future research is needed to compare the long-term outcomes of SL and robot-assisted technologies.

## Acknowledgments

Thanks to the first People’s Hospital of Xiaoshan District for providing the surgery platform.

## Author contributions

**Conceptualization:** Zhengwei Lai.

**Data curation:** Jin Zhao, Zhengwei Lai.

**Formal analysis:** Jin Zhao, Zhengwei Lai.

**Funding acquisition:** Jin Zhao, Zhengwei Lai.

**Investigation:** Zhengwei Lai.

**Methodology:** Zhengwei Lai.

**Project administration:** Zhengwei Lai.

**Resources:** Jin Zhao, Zhengwei Lai.

**Software:** Zhengwei Lai.

**Supervision:** Jun Wu, Jin Zhao, Zhengwei Lai.

**Validation:** Jun Wu.

**Visualization:** Jun Wu.

**Writing – review & editing:** Jun Wu, Jin Dong.

**Writing – original draft:** Jin Dong.
